# Perceptions of COVID-19 Mitigation Strategies between Rural and Non-Rural Adults in the US: How Public Health Nurses Can Fill the Gap

**DOI:** 10.3390/nursrep12010019

**Published:** 2022-03-02

**Authors:** Alan M. Beck, Amy J. Piontek, Eric M. Wiedenman, Amanda Gilbert

**Affiliations:** 1 Prevention Research Center, Washington University in St. Louis, St. Louis, MO 63130, USA; ericw@wustl.edu (E.M.W.); a.s.gilbert@wustl.edu (A.G.); 2 Goldfarb School of Nursing, Barnes-Jewish College, St. Louis, MO 63110, USA; amy.piontek@barnesjewishcollege.edu; 3 Department of Surgery, Division of Public Health Sciences, Washington University in St. Louis, St. Louis, MO 63130, USA

**Keywords:** rural, COVID-19, public health nursing

## Abstract

The purpose of this study was to capture the perceptions of COVID-19 mitigations’ efficacy of rural and non-rural participants, using the health belief model (HBM), as well as to describe where public health nursing may be able to fill behavior gaps in rural communities. Rural and non-rural participants completed electronic surveys. Surveys collected demographic information and perceptions of various mitigation strategies’ effectiveness. Rurality was significantly associated with perceptions of the effectiveness of public health mitigation strategies including wearing facemasks, limiting time indoors, avoiding gatherings, non-essential business closure, and staying home. Our findings suggest people in rural areas perceive mitigations to be effective. Other researchers have consistently shown rural residents are least likely to partake in the same mitigations. Rural public health nurses on the front line serve as the key to closing the aforementioned gap. Understanding where their community’s perceptions lie is pivotal in creating educational programs to continue mitigation efforts as we embark on the second year of this pandemic.

## 1. Introduction

At the onset of the SARS-CoV-2 (COVID-19) pandemic in the United States (US), metropolitan areas were the most highly impacted by the infection [1]. Rural areas were thought to have some protection from the virus inherently due to their sparse nature. Over time, however, this perceived protection of rural areas dwindled, thereby becoming the US’s newest hot spot [1,2]. Unfortunately, as has been the normal trajectory with COVID-19, by September 2020 death rates in rural areas surpassed those in urban areas [1,3]. In fact, rural areas have been hit harder; roughly 1 in 434 rural Americans have died of COVID, compared to 1 in 513 urban Americans [4].

Rural local health departments (LHD) are key resources in their respective communities. Unfortunately, LHDs in rural areas are among the most understaffed and underfunded health departments in the nation [5,6]. While LHDs in rural areas serve smaller populations, these communities tend to have limited access to medical care [7,8], poor health outcomes [7,8,9], and experience health disparities related to risky health behaviors [7,10,11]. Leadership at most LHDs in metropolitan areas tends to be someone with a formal degree in public health [6]; in contrast, rural LHDs are three times as likely to be led by someone with a nursing degree [12,13,14]. Nurses are the linchpin that holds rural health departments together, especially considering rural LHDs frequently offer more direct clinical services [15,16]. Unfortunately, prior to the COVID-19 pandemic, there was an estimated 36% decrease in staffing of public health nurses [17]. Therefore, rural area LHDs were likely the most impacted given much of their staff are nurses. This amalgamation of the need for public health nurses in rural areas, services provided by rural LHDs, and a global pandemic, left rural LHDs at risk of being overwhelmed—their communities would need to do their part to protect themselves.

Practices such as avoiding contact with others who are ill, social distancing, covering the nose and mouth when coughing or sneezing, washing hands or using approved hand sanitizer, and using face coverings when in public places are all recommendations established by the Centers for Disease Control and Prevention (CDC) to reduce the spread of COVID-19 [18]. It is also recommended that when a person falls ill with COVID-19-like symptoms that they remain quarantined and away from others [19]. It has been well documented that mitigation strategies decrease the spread of COVID-19 in community and healthcare settings [20,21,22]. However, these strategies are only effective if consistently followed.

Overall, Americans report agreement with public health mitigations; though, few of the respondents in these studies were from rural areas [23,24,25]. There is hope now with the approval of vaccines; however, there is still a concern for variants coupled with vaccine hesitancy. Rural areas, and states with large rural populations, are demonstrating some of the lowest vaccine rates in the nation [26]. The coalescing of low vaccination rates, rural populations’ increased vulnerability to serious infection, increased vaccine hesitancy, and the concern for variants, means public health mitigation strategies will likely continue to be warranted [3,27]. Due to the likely need for ongoing mitigation strategies, the present study was designed to capture the perceptions of mitigation strategies among a sample of rural residents as compared to non-rural residents.

## 2. Materials and Methods

Participants were recruited from social media posts (e.g., Facebook, Twitter) and email list serves from 26 August to 17 September 2020. An image, with a link imbedded, describing the study was posted to social media. (Example post, “If you live in a rural area, please take our survey for a chance to win an Amazon gift card!”) Various local, regional, and national advocacy groups distributed social media posts and emails. Emails contained a brief description of the study with a link to the survey’s consent form. Social media posts contained a link embedded in an image describing the study—when the potential participant clicked the image, they were routed to the consent form. If potential participants agreed, they were then taken to the survey. All responses were anonymous unless they wanted to be entered into the raffle for a gift card in which the participant would provide contact information. Inclusion criteria for participation were ≥18 years of age and the ability to read English. All data were collected in Qualtrics software [28]. The Washington University in St. Louis Institutional Review Board approved the study with an exempt status.

### 2.1. Theoretical Framework

The health belief model (HBM) first developed by Godfrey H. Hochbaum in the late 1950s is the theoretical framework supporting this study. Hochbaum developed the HBM to understand peoples’ behaviors associated with their perceived susceptibility in contracting disease, perceived severity of the disease, perceived benefits to reducing the threat of disease, and perceived barriers to action in decreasing the risk of disease [29]. The first three constructs of perceived susceptibility, perceived severity, and perceived benefits were measured in this study.

### 2.2. Measures

#### 2.2.1. Demographics

Participants were asked to provide their gender (male, female), age, education level (less than high school, some college/associates degree, college, more than college), race (black or African American, white, American Indian or Alaska Native, Asian, Native Hawaiian, or Pacific Islander), ethnicity (Hispanic), marital status (married, widowed, divorced, separated, never married, unmarried couple), household income (less than 20,000, 20,000–49,999, 50,000–79,999, 80,000 or more), and zip code. In order to minimize the number of variables, educational level was used as a surrogate to income as they were highly correlated. To define rurality, each participant’s zip code was compared to the Rural-Urban Continuum Codes (RUCC) [30]—a code of four or higher was operationally defined as rural [31,32].

#### 2.2.2. Impact of COVID-19

Participants were asked how COVID-19 influenced one’s personal daily life for each of the following five domains (work, school, finances, physical health, and mental health). Five response options were provided ranging from not at all, a little, a moderate amount, a lot, to a great deal. Responses were collapsed into three categories, not at all (not at all), somewhat (a little, a moderate amount), and a lot (a lot, a great deal) for analysis.

#### 2.2.3. COVID-19 Worries

Participants were asked about their worries regarding the pandemic (e.g., contracting COVID-19, transmitting COVID-19 to someone else, family/friends contracting COVID-19, having enough food, and loss of income). Five response options were provided ranging from not at all, a little, a moderate amount, a lot, to a great deal. Responses were collapsed into three categories, not at all (not at all), somewhat (a little, a moderate amount), and a lot (a lot, a great deal) for analyses.

#### 2.2.4. Mitigation Strategies

Participants were asked about COVID-19 mitigation strategies for both individual behaviors (wearing of a facemask, consulting a health care provider if you feel sick, avoiding/limiting indoor public spaces, avoiding outdoor spaces, avoiding large gatherings, avoiding contact with people at high risk, and limiting errands requiring public places), and public health measures (closure of schools, closures of all shops not considered essential, non-essential workers stay home, and people over the age of 70 stay home). Five response options were provided ranging from not at all effective, slightly effective, moderately effective, very effective, to extremely effective. Responses were collapsed into three categories, not at all effective (not effective at all), moderately effective (slightly effective, moderately effective), and highly effective (very effective, extremely effective) for analysis.

### 2.3. Analysis

Quantitative data analysis was conducted using SPSS (version 26) (IBM Corporation. Armonk, NY, USA). The dependent variables were participant perceptions of the impact of COVID-19, COVID-19 worry, and perceptions of the effectiveness of COVID-19 mitigation strategies. The independent variable was rurality based on the RUCC and dichotomized into rural and urban. Descriptive statistics, frequency tables, and chi-squared analyses were conducted. Assumptions of sample size were met for chi-squared analyses.

## 3. Results

In total, 278 respondents completed the survey (Table 1). Fifty percent of participants were classified as rural and 50% as non-rural. Most participants were female (88%), white (96.7%), married (71%), and between the ages of thirty-six to sixty years old (61%). About half of the participants reported a household income of USD 80,000 or greater and around 60% had a college degree or higher. Among rural participants, 79% were female, 99% white, and 62% were between the ages of thirty-six and sixty. In regard to household income and marital status, 46% of rural participants reported an annual household income equal to or greater than USD 80,000 and 70% reported being married. Around 11% of rural participants had a high school degree or GED, with most (43%) completing some college or associates degree. Compared to rural participants, there were a higher percentage of non-rural participants who reported being female (87%), earning USD 80,000 or more (58%), having more than a college degree (41%) and being married (73%) and a lower percentage of non-rural participants reporting being white (94%), and aged between thirty-six and sixty years old (60%).

### 3.1. Perceptions on the Impact of COVID-19 on Daily Life

Regarding the impact of the COVID-19 pandemic on work, 87% of the total sample reported the COVID-19 pandemic negatively impacted work some (53%) or a lot (34%), and 14% reported no impact at all. When asked about the financial impact of COVID-19, 66% reported being negatively impacted some (52%) or a lot (14%), and 87% reported being worried some (24%) or a lot (63%), about the potential for income loss. In terms of the negative impact of COVID-19 on health, 65% reported their physical health was negatively impacted some (53%) or a lot (12%) by the COVID-19 pandemic and 87% reported their mental health was negatively impacted some (53%) or a lot (34%). No significant correlations were found between rurality and COVID-19 impact.

### 3.2. Participant Worries Regarding COVID-19

Overall, 85% of participants worried some (48%) or a lot (37%) about getting COVID-19 and 78% worried some (39%) or a lot (39%) about giving COVID-19 to others. The majority of participants (97%) reported being worried some (18%) or a lot (79%) about having enough food and most (87%) reported being worried some (24%) or a lot (63%) about income loss. Only worry about family or friends getting COVID-19 was significantly correlated with rurality. (x^2^(2) = 7.687, *p* = 0.021) Among rural participants, 80% reported being worried some (52%) or a lot (28%) about family and friends getting COVID-19, while 67% of non-rural participants worried some (50%) or a lot (17%) about family and friends getting COVID-19.

### 3.3. Perceptions on the Effectiveness of Individual Behaviors for Staying Safe from COVID-19

We found significant correlations between rurality and perceptions of effectiveness for the behaviors of wearing a face mask (x^2^(2) = 9.997, *p* = 0.007), limiting time spent indoors in public spaces (x^2^(2) = 13.903, *p* = 0.001), avoiding large gatherings (x^2^(2) = 10.006, *p* = 0.007), and limiting the frequency of necessary errands (x^2^(2) = 9.015, *p* = 0.011). When asked whether wearing a facemask was “effective for keeping you safe from COVID-19”, 82% of rural participants reported wearing a facemask was moderately (39%) or highly (43%) effective for protecting against COVID-19, while 75% of non-rural participants reported a facemask was moderately (50%) or highly (25%) effective. When asked about limiting time spent indoors in public spaces, 69% of rural participants perceived this behavior as moderately (42%) or highly (27%) effective for keeping them safe from COVID-19. Among non-rural participants, 55% perceived this behavior as moderately (45%) or highly (10%) effective. In regards to avoiding large gatherings, 68% of rural participants perceived this behavior as moderately (40%) or highly (28%) effective, while 56% of non-rural participants perceived this behavior as moderately (43%) or highly (13%) effective. Among rural participants, 77% reported “limiting frequency of necessary errands requiring public places (e.g., grocery shopping)” was moderately (50%) or highly (27%) effective at keeping them safe from COVID-19, while 65% of non-rural participants reported this behavior as moderately (51%) or highly (14%) effective. We did not find significant associations between rurality and perceptions on the effectiveness of consulting a health care provider, avoiding outdoor public spaces, and avoiding contact with at-risk populations.

### 3.4. Perception on the Effectiveness of Public Health Measures for Preventing COVID-19

When assessing participant perceptions for the effectiveness of public health measures to mitigate COVID-19 transmission, we found significant correlations between rurality and public health measures for guidelines recommending the wearing of facemasks (x^2^(2) = 16.486, *p* < 0.001), closing non-essential businesses (x^2^(2) = 14.324, *p* = 0.001), recommending people aged 70 and over or with a medical condition stay at home except for essential needs (x^2^(2) = 9.344, *p* = 0.009), and for non-essential workers to stay at home except to do basic shopping or because urgent medical care is required (x^2^(2) = 13.116, *p* = 0.001). For guidelines recommending the wearing of face masks, 78% of rural participants perceived this public health measure as moderately (33%) or highly effective (45%), while 62% of non-rural participants viewed this public health measure as moderately (39%) or highly (23%) effective. When asked about the closure of non-essential businesses, 91% of rural participants viewed this measure as moderately (29%) or highly (62%) effective, while 88% of non-rural participants reported this public health measure was moderately (49%) or highly (39%) effective. Among rural participants, 73% felt recommendations for those aged 70 and older or with a medical condition to stay home were moderately (48%) or highly (25%) effective. Among non-rural participants, 63% reported this was a moderately (52%) or highly (11%) effective public health measure. Seventy-nine percent of rural participants perceived public health measures recommending non-essential workers stay at home as moderately (38%) or highly (48%) effective. Among non-rural participants, 77% felt this was a moderately (50%) or highly (27%) effective public health measure for mitigating the spread of COVID-19. We did not find significant correlations between rurality and perceptions on the effectiveness of the public health measures of closing schools.

## 4. Discussion

Rurality was significantly associated with perceptions of the effectiveness of public health mitigation strategies including the wear of facemasks, limiting time indoors, avoiding large gatherings, closing non-essential businesses, and elderly and non-essential workers staying home unless required to go out. Further, rural areas demonstrated more concern for the wellbeing of family and friends compared to more urban areas. There appears to be a paradox whereby our findings suggest rural people think mitigations are effective; however, they may not actually partake in the same mitigations they deem effective. Prior research has found rural communities may not adhere to mitigations at the same rate as their urban counterparts [24,33,34,35]. There may be a disconnect between rural residents’ perceptions and risk. Perhaps they deem the risk low since rural areas are dispersed, or perhaps it is related to social media misinformation [36]. Whatever the case may be, public health nurses may be the answer [37].

The higher the percentage of people reporting COVID-19 negatively impacting their work, financial security, mental health, physical health, and having enough food, the higher their perceived impact (severity) of the disease. In this instance, it may not be the severity of the disease process itself, but rather the implications surrounding being ill with the disease (i.e., not being able to work or loss of job). Those participants with higher percentages of worry related to contracting and transmitting COVID-19 correlated with an increased perception of susceptibility to the disease. In other words, they perceived themselves to be susceptible to contracting or transmitting the disease to others. The assessment finding related to the participants’ perceptions of the effectiveness of individual behaviors for staying safe from COVID-19 parallels the HBMs construct of perceived benefits to the mitigation strategies. Those participants who reported specific mitigation strategies to be effective in curving the spread of the disease were essentially indicating these actions produced benefits in controlling the spread of COVID-19.

Historically, the role of the public health nurse may not have been well understood, until the world was faced with one of the deadliest pandemics in the last 100 years. Public health nurses began doing what they do best by working collaboratively with community partners to educate people on what they can do to control the spread of COVID-19 [38]. Communities learned what they needed to do to protect themselves and others from this disease. Once vaccines became available, public health nurses provided education to community partners in the efficacy of vaccines, proper storage of vaccines, and strategies to track multi-dose vaccine administration. Public health nurses continue to work with communities for vaccine administration and provide ongoing support and education for those still skeptical of the safety of the vaccines. The role of the public health nurse in conjunction with LHDs is to empower people through education. At a time of great uncertainty, one thing is clear—RNs who work within communities have the knowledge and skills to deliver factual and relevant information regarding mitigation strategies [38]. They are the backbone to addressing this nation’s need to eradicate COVID-19. LHD nurses could be the link between perceptions of mitigation success and actual behaviors.

Local health departments and public health nurses are often well connected to the available resources in the community and can act as resource hubs for community members. As our study findings suggest, and previous studies have also reported [39,40], many individuals living in rural areas are concerned about not having enough food and income loss throughout the COVID-19 pandemic. Many LHDs maintain current community resource guides including local food pantries, income, utility, transportation, housing assistance programs, and many other social services. Public health nurses and LHDs can leverage their position and connection to these resources to help community members connect to the services they need.

As COVID-19 continues to be a public health crisis in the United States, and with vaccine rates continuing to lag in rural areas and states with large rural populations, findings from this study may provide opportunities to emphasize rural communities’ concern for the well-being of others to address vaccine hesitancy. Effective COVID-19 immunization programs are evidence based [27], and previous studies have identified emphasizing the personal risks of failing to get the vaccine and the potential spread to others in the community as effective health communication messaging [41,42]. Further, researchers suggest tailoring messaging to appeal to the opinions and values of a sub-population can increase their effectiveness [43,44,45]. Therefore, we provide the following recommendations for messaging aimed at rural communities in the United States:

1. Highlight how getting vaccinated and mitigations can help protect your family and friends from becoming sick [46], and a social responsibility to help your community [40].
2. Continue to discuss the importance of wearing a face mask to prevent the spread of COVID-19. Suggest or encourage outdoor gatherings.
3. Messaging that is from the local community, about the local community (e.g., public health nurses) [47]. Emphasize public health nurses and local health departments as valuable resources in the community, both for answering questions about COVID-19 and for their connections to community resources (food pantries/banks, utility assistance programs, SNAP, WIC, etc.).

### Limitations

One limitation of the study was the cross-sectional nature in which data were collected; therefore, the results are correlational and causation cannot be determined. A second limitation was the relatively small sample size. A third limitation was that the sample was relatively small and a majority of white females who have reported higher threat perceptions of COVID-19 [46,48]. Although perceived barriers to COVID-19 transmission mitigation strategies were not assessed, future studies should be explored using this construct to gain a greater understanding to address these barriers for greater adherence to disease prevention. Using a qualitative approach to understanding the barriers could be beneficial in understanding specific communities’ unique situations to reduce or eliminate recognized barriers. Another limitation to the current study was the fact that rural communities here had abnormally high income—likely due to the recruitment methodology (i.e., online). Lastly, we did not survey people about their actual behavior, only their perceptions of the effectiveness of mitigation strategies.

## 5. Conclusions

For decades, public health professionals have relied on and continue to use the HBM to understand peoples’ health behaviors. Whether it is receiving the COVID-19 vaccine and booster or wearing a mask in public places, understanding the public’s view in their susceptibility of contracting the disease, the severity the disease will have on their personal lives, and the value placed on mitigation strategies, greatly assists public health nurses and health departments. They are able to align their educational and resource outreach programs to those who may have low perceptions of the severity COVID-19 can have on their lives, their low perceptions of the susceptibility they may have in contracting or spreading the disease, and most importantly, those who may not perceive the mitigation strategies to be beneficial to themselves and those in their family or community. In this study, we found that rural people consider mitigations strategies more effective as compared to their urban/suburban counterparts. While other studies have found rural areas tend to partake in mitigation strategies at a lower clip, we posit those same rural areas might still believe in the efficacy of the strategies. One under-resourced yet passionate messenger to close the gap between mitigation perceptions and partaking in the mitigation strategy is the local public health nurse. Public health nurses have a plethora of information as well as the passion to serve their communities. Understanding their community’s perceptions of COVID-19 is the first step in addressing the ongoing need for further education and disease prevention strategies. Finally, after years of divestment, it is recommended to bolster funding to rural LHD’s in order to hire more RN’s. LHD RN’s in rural areas are the lifeblood and key to success in navigating the pandemic now and in the future.

## Figures and Tables

**Table 1:** Demographic characteristics of rural and non-rural respondents.

	Total (*n* = 278)	Rural (*n* = 139)	Non-Rural (*n* = 139)
Female, *n* (%)	245 (88.1)	125 (78.51)	120 (87.0)
Race, *n* (%)			
White	265 (96.7)	135 (99.3)	130 (94.2)
Black	6 (2.2)	1 (0.7)	5 (3.6)
Asian	2 (0.7)	0 (0.0)	2 (1.4)
American Indian or Alaska Native	1 (0.4)	0 (0.0)	1 (0.7)
Hispanic/Latino, *n* (%)	1 (0.4)	1 (0.7)	0 (0.0)
Age, *n* (%)			
35 and younger	60 (22.5)	27 (20.3)	33 (24.6)
36–60 years	163 (61.0)	83 (62.4)	80 (59.7)
61 and older	44 (16.5)	23 (17.3)	21 (15.7)
Income, *n* (%)			
Less than USD 20,000	13 (4.8)	9 (6.6)	4 (3.0)
USD 20,000–49,999	52 (19.3)	33 (24.3)	19 (14.3)
USD 50,000–79,999	64 (23.8)	31 (22.8)	33 (24.8)
USD 80,000 or more	140 (52.0)	63 (46.3)	77 (57.9)
Education, *n* (%)			
High school/GED	20 (7.2)	15 (10.8)	5 (3.6)
Some college/associates degree	87 (31.4)	60 (43.2)	27 (19.6)
College	91 (32.9)	41 (29.5)	50 (36.2)
More than college	79 (28.5)	23 (16.5)	56 (40.6)
Marital Status, *n* (%)			
Married	197 (71.4)	97 (69.8)	100 (73.0)
Widowed	5 (1.8)	4 (2.9)	1 (0.7)
Divorced	31 (11.2)	21 (15.1)	10 (7.3)
Separated	4 (1.4)	2 (1.4)	2 (1.5)
Never married	17 (6.2)	6 (4.3)	11 (8.0)
Unmarried couple	22 (8.0)	9 (6.5)	13 (9.5)

## Data Availability

The data are not publicly available due to privacy concerns.
